# Investigation of bentonite–goethite mixture as a novel material for low-level landfill liners

**DOI:** 10.1038/s41598-025-31747-y

**Published:** 2025-12-20

**Authors:** Mohammad Nadi, Amin Azhari, Hajar Share Isfahani, Hem B. Motra, Mahmoud Hefny, Abbas Salati, Mohsen Bazargan

**Affiliations:** 1 Department of Mining Engineering, Isfahan University of Technology, Isfahan, 84156-8311 Iran; 2 Escuela de Ingeniería Química, Pontificia Universidad Católica de Valparaíso, Av. Brasil 2162, Valparaíso, 2340025 Chile; 3 Institute of Geosciences, Marine and Land Geomechanics and Geotechnics, Christian-Albrechts-Universität, 24118 Kiel, Germany; 4 Department of Earth and Planetary Sciences, Geothermal Energy and Geofluids, ETH Zurich, Zurich, Switzerland; 5 Geology Department, Faculty of Science, Qena University, Qena, 83523 Egypt; 6 Department of Civil Engineering, Isfahan University of Technology, Isfahan, 84156-83111 Iran; 7 Rock Engineering, Tyrens AB, Stockholm, Sweden; 8 Department of Earth Sciences, Uppsala University, Villavägen 16, 752 36 Uppsala, Sweden

**Keywords:** Energy science and technology, Engineering, Environmental sciences, Materials science, Solid Earth sciences

## Abstract

The safe containment of hazardous waste requires landfill liner materials with both effective radiation shielding and strong hydro-mechanical performance. This study investigates the potential of a bentonite-goethite mixture as a novel material for hazardous waste landfill liners. The study examines the radiation shielding of the mixtures, represented by the linear attenuation coefficient, through experimental (Na (Tl) spectrometer detector), numerical (MCNP code), and reference database (XCOM and PHY-X) approaches. Moreover, the hydraulic permeability and mechanical properties are evaluated experimentally. For this, the varying proportions of goethite from 10 to 50% were examined. The results show an increase of up to 20, 24, and 28 percent in the linear attenuation coefficient at gamma ray energies of Cs$^{137}$ (661.6 keV) and Co$^{60}$ (1173.2 and 1332.5 keV). Higher goethite percentages, correlating with density variations and enhancing radiation shielding effectiveness. Numerical and reference database results align closely with experimental findings, suggesting their utility for assessing other mixtures. The direct shear test reveals that with an increase of goethite proportion to 50 percent, the cohesion is reduced to half and the friction angle is inclined twice the pure bentonite values, attributed to bentonite reduction and goethite roughness. Unconfined compressive strength trends show 20% improvement at specific mixture composition with 30% goethite, while hydraulic conductivity inclines with goethite content to $\mathrm { 8.8\times 10^{-10}}$ m/s. In this study the bentonite-goethite mixture illustrates improving radiation shielding and maintaining hydro-mechanical properties for landfill liners. This may offer a sustainable alternative using waste materials from mineral processing, contributing to waste management and environmental sustainability.

## Introduction

Landfills are geo-environmental structures that play a vital role in the management of low-level radioactive waste. These systems pose two major environmental risks: potential gamma-ray radiation and the leaching of hazardous materials into surrounding soil and water sources^1–3^. To mitigate these risks, effective radiation shielding and hydraulic impermeability are essential, with landfill liners serving as the primary barrier. Clay soil is widely used as a base material for landfill liners due to its availability, durability, and relatively low permeability. Recent studies have focused on enhancing the radiation shielding and hydro-mechanical performance of clay soil liners^4–8^.

According to radiation theory, density is the key factor influencing gamma-ray attenuation, as represented by the linear attenuation coefficient ($\mu$)^4,9–14^. This principle is widely applied in geotechnical and geomechanical investigations^15,16^. Consequently, the incorporation of heavyweight additives—such as lead, steel slag, barite, zeolite, magnetite, and hematite—into clay liners has emerged as a promising strategy to improve radiation shielding^17–20^. Regarding sustainable development, it is desirable to use high-density industrial by-products as additives in bentonite-based liners to enhance the radiation shielding performance.

Isfahani and Azhari (2021) evaluated the effect of basalt fiber additions (0.5%, 1%, 2%, and 5%) on the radiation shielding performance of clay soil^21^. Their results demonstrated that basalt fiber enhanced shielding efficiency, with 2% addition yielding the highest linear attenuation coefficients of 12.3 m$^{-1}$, 10.14 m$^{-1}$, and 8.5 m$^{-1}$ for Cs-137 (661.6 keV) and Co-60 (1173.2 and 1332.5 keV), respectively. Elsafi et al. (2022) experimentally determined the linear attenuation coefficients of prepared samples using a Na(Tl) detector and four radioactive point sources (Am-241, Cs-137, Co-60, and Eu-152)^22^. Their study also introduced additional radiation protection metrics, including average cost layer, average independent path, and radiation defense efficiency. Results indicated that bentonite–iron composites exhibited superior shielding efficiency compared to bentonite–ferrosilicon composites at low and intermediate gamma energies.

Isfahani et al. (2019) investigated the addition of 10–40% steel slag to bentonite clay to enhance radiation shielding^19^. They found that steel slag significantly improved the linear attenuation coefficient, with a 40% addition offering substantial protection while maintaining permeability within acceptable landfill cover limits. textcolorblueFalahi et al. investigated the enhancement of bentonite clay with magnetite powder (15%, 30%, 45%) to improve radiation shielding and gas permeability for radioactive waste management^23^ . The 30% magnetite sample (B70-M30) significantly improved radiation shielding (up to 62%) and reduced gas permeability by 73.4% under wet-dry cycles. Despite some permeability increase over time due to micro-cracking, B70-M30 showed the best overall performance, highlighting its potential as a durable barrier material. Another study developed a gamma-ray shielding material by combining mine waste, bentonite, and cement. The material, containing 55% cement and 17% mine waste, effectively reduced low-energy gamma radiation with a thickness of less than 1 cm. The results suggest that mine waste composites offer a sustainable, cost-effective solution for radiation shielding and eco-friendly construction^24^.

Beyond radiation shielding, the hydro-mechanical performance of liner materials is critical. Devarangadi and Masilamani (2023) examined the mechanical properties of granulated blast furnace slag mixed with bentonite for landfill liners^25^. Their findings revealed that unconfined compressive strength (UCS) increased with higher slag content and longer curing periods, while hydraulic conductivity values were 1,000 to 10,000 times lower than the required threshold. Kim and Kim (2017) studied cement-stabilized bentonite–sand mixtures, incorporating small amounts of fibers and metakaolin to enhance strength^26^. The optimal mixture with 10% bentonite and 1% each of fiber and metakaolin showed UCS improvements of approximately 40% and 70%, respectively.

Firoozfar and Khosroshiri (2016) conducted laboratory tests on pure clay and lime-treated samples^27^. Their results indicated that lime and bentonite treatments reduced hydraulic conductivity to the order of 10$^{-8}$ m/s, meeting Environmental Protection Agency (EPA) standards. Compressive strength decreased with increasing bentonite but increased significantly with lime addition, with 1% lime yielding a 75% increase in strength. Falamaki et al. (2018) evaluated the hydro-mechanical behavior of sandy soil and bentonite clay stabilized with dicalcium phosphate (DCP) to immobilize heavy metals^28^. Their study showed that adding 0.2% DCP improved shear strain at peak strength and reduced permeability from 10$^{-4}$ m/s to below 10$^{-9}$ m/s. Based on these findings, a multi-layer compacted clay liner incorporating DCP-treated soil is recommended for enhanced heavy metal stabilization. Abhinav (2025) evaluated bentonite-paste tailings (BPT) mixtures as sustainable barrier materials for waste containment. Tests showed that BPT offers low hydraulic conductivity and improved shear strength with increasing bentonite content (up to 4%)^29^. However, higher bentonite levels also increased desiccation cracking, posing a risk to barrier integrity. The findings emphasize the need to balance strength and crack resistance when optimizing BPT mixtures for long-term environmental protection.

Despite extensive research on bentonite-based liners enhanced with additives such as steel slag, basalt fibers, and magnetite, no comprehensive study has investigated the use of mine waste goethite—a dense, iron-rich mineral—as a multifunctional additive for low-level radioactive waste containment. Specifically, the combined assessment of hydraulic and gas permeability, mechanical performance, and gamma-ray shielding efficiency of bentonite–goethite mixtures remains unexplored. Existing studies often evaluate these parameters in isolation, neglecting the trade-offs between improved radiation attenuation and potential increases in permeability, as well as mechanical implications under landfill-relevant conditions. These gaps underscore the need for an integrated, multi-criteria performance evaluation of bentonite–goethite liners.

## Materials and methods

### Materials, sample preparation and characterization

**Fig. 1:**
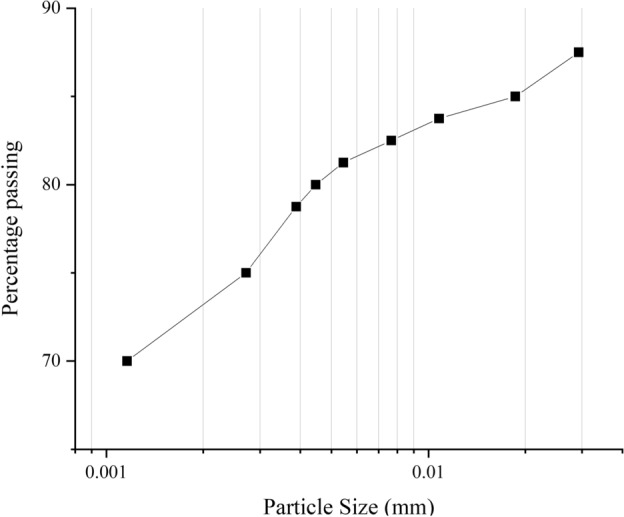
The particle grading curve of utilized sodium-bentonite.

The present study employs Bentonite clay as the base material along with goethite powder as a heavyweight additive. Bentonite clay, containing over 95% montmorillonite minerals, is a relatively impermeable soil commonly employed in landfill cover layers^30,31^. The sodium-based bentonite used in this investigation is characterized by the grading curve illustrated in Fig. 1 according to ASTM D422-63. The figure indicates that the grain sizes are predominantly less than 0.075 mm, with a density of 2.65 g/cm$^3$. The grain size distribution curve indicates that bentonite is predominantly composed of ultra-fine particles, with most sizes falling below 0.02 mm. Approximately 70% of the particles are smaller than 0.002 mm, confirming a high proportion of clay-sized fractions. The percentage passing increases steadily with particle size, reaching nearly 89% at 0.02 mm, with a notable steep rise between 0.002 mm and 0.005 mm, reflecting the concentration of particles in this very fine range. This fine texture is characteristic of bentonite and underpins its high surface area, swelling potential, and low hydraulic conductivity.

**Fig. 2:**
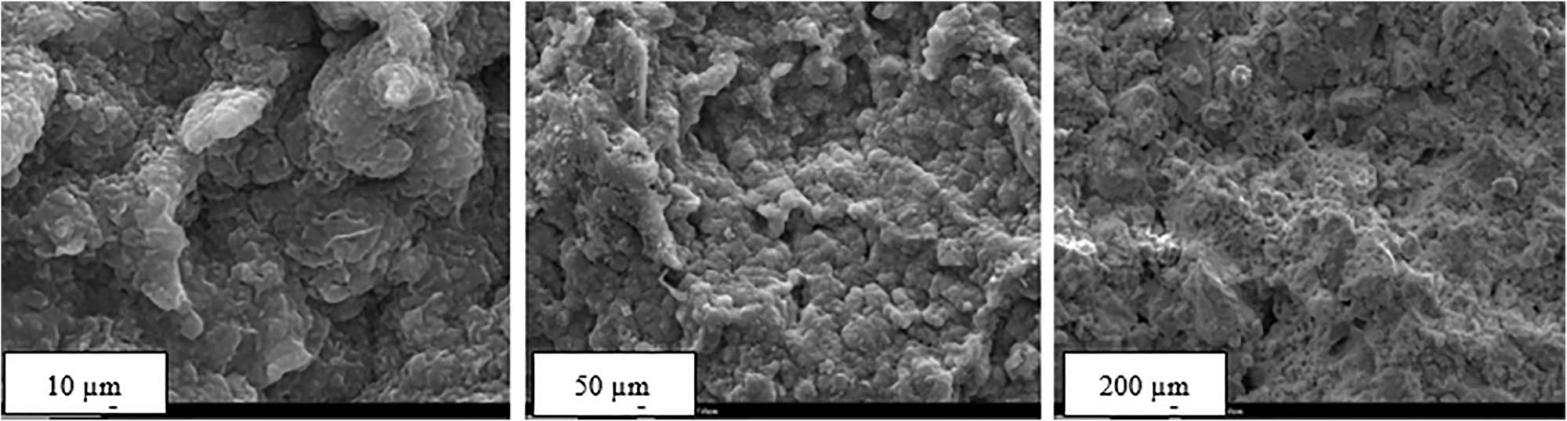
Scanning electron microscopy (SEM) images of sodium bentonite at three scales.

To better understand the behavior of the studied mixtures, the recognition of the mixture components is always of great importance. Therefore, in this study, X-ray fluorescence (XRF) spectroscopy tests were used to analyze the present elements, and scanning electron microscopy (SEM) imaging was used to examine the particle structure. Table 1 presents the atomic and mass percentage of the used sodium bentonite from XRF analysis, and Fig. 2 illustrates the scanning electron microscopy (SEM) images of sodium bentonite in three scales. The data presented in Table 3 have a good agreement with the previous studies^32,33^. The SEM images of sodium bentonite at 10, 50, and 200 $\upmu$m scales show its characteristic platy particle morphology and hierarchical porosity. At the 10 $\upmu$m scale, closely packed, sheet-like clay platelets with sizes from sub-micron to a few micrometers dominate, exhibiting minimal visible voids. At 50 $\upmu$m, these particles form rough-surfaced aggregates with small interparticle pores, indicating moderate porosity. At 200 $\upmu$m, the structure reveals a network of aggregated clusters with larger, interconnected voids, highlighting the transition from dense particle packing at the micro-scale to more pronounced macro-scale porosity.

**Table 1:** Atomic and weight percentage of the used sodium bentonite from XRF analysis.

Element	Atomic percentage	Weight percentage
O	44.38	30.17
Si	19.23	22.94
Na	15.21	14.86
Cl	11.10	16.72
Ca	4.16	7.08
Al	3.87	4.43
Fe	1.25	2.97
Mg	0.81	0.83

**Fig. 3:**
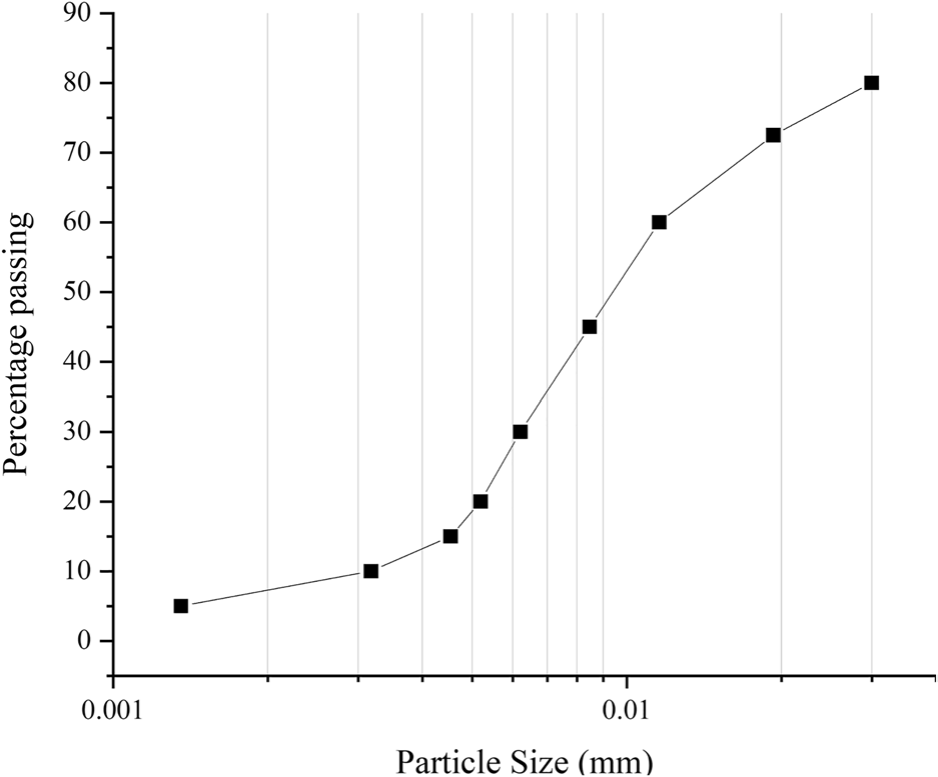
Grain size distribution of the used goethite.

**Fig. 4:**
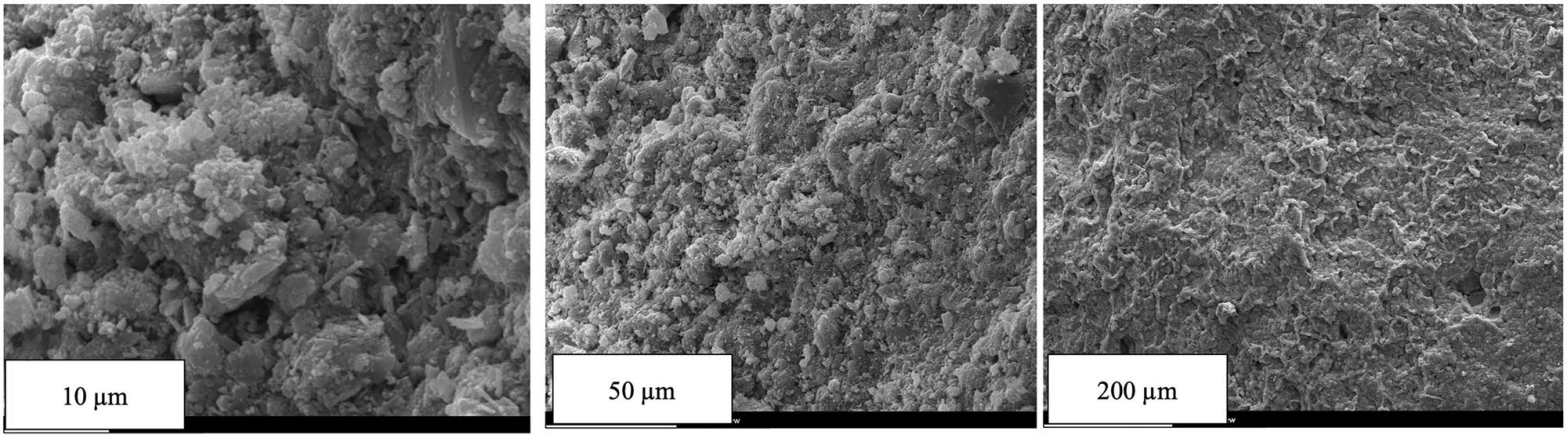
Scanning electron microscopy (SEM) images of the used goethite at three scales.

Goethite is a waste generated by removing iron from the zinc sulfate solution generated by leaching calcine with sulfuric acid. For every one ton of iron ore concentrate produced, approximately 2.5–3.0 tons of iron ore tailings will be discharged. Statistics show that there are 130 million tons of iron ore tailings discharged every year^34^. In 2023, Iran produced approximately 88 million metric tons of iron ore, up from about 68 million metric tons in 2018, and as one of the largest producers in the Middle East, in which, its six large open-pit mines generate a significant amount of goethite as waste material. In this study, goethite is used as the additive in the mixtures with a density of 4.3 g/cm$^3$. Figure 3 depicts the grain size distribution of the implemented goethite, where about 40 percent of the particles are finer than 0.075 mm. Figure 4 shows the SEM image of goethite powder in three scales. These images can identify the microstructure and the degree of porosity of goethite particles. Table 2 presents the percentage of elements in the used goethite, which shows that oxygen, iron, silicon, and calcium account for more than 90% of the main elements in this material. Where iron with a weight percentage of about 42% and a relatively high atomic percentage plays a fundamental role in absorbing gamma rays. Moreover, the Atterberg limits and the PH values of the implemented bentonite and Goethite are presented in Table 3.

**Table 2:** Atomic and mass percentage of the used goethite from XRF analysis.

Element	Atomic percentage	Mass percentage
O	47.06	25.44
Fe	22.59	42.62
Ca	10.66	14.43
Si	10.35	9.82
Na	5.54	4.30
Al	2.76	2.52
Mg	0.99	0.81
Cl	0.04	0.05

**Table 3:** The Atterberg limits and PH values of the utilized bentonite and geothite.

Property	Bentonite	Goethite
Liquid Limit (LL)	167%	40%
Plastic Limit (PL)	53%	20%
Plasticity Index (PI)	114%	10%
Shrinkage Limit	10%	15%
pH	8	5

The preparation of mixtures containing bentonite and varying proportions of goethite powder, namely B90G10 (10% goethite), B80G20 (20% goethite), B70G30 (30% goethite), B60G40 (40% goethite), and B50G50 (50% goethite), has been considered.

To prepare identical and comparable specimens, all were fabricated at their respective optimum moisture content. For this purpose, the optimum moisture content and corresponding density of each mixture were determined via the standard proctor compaction test. The compaction tests were conducted in accordance with ASTM D698. A compactive effort of 600 $\mathrm {kN \, m/m^3}$ was applied using a 4-inch mold in three layers, each compacted with 25 blows from a 2.5 kg rammer dropped from a height of 305 mm. After compaction, specimens were sealed and allowed to equilibrate for up to 24 h for moisture conditioning.

**Fig. 5:**
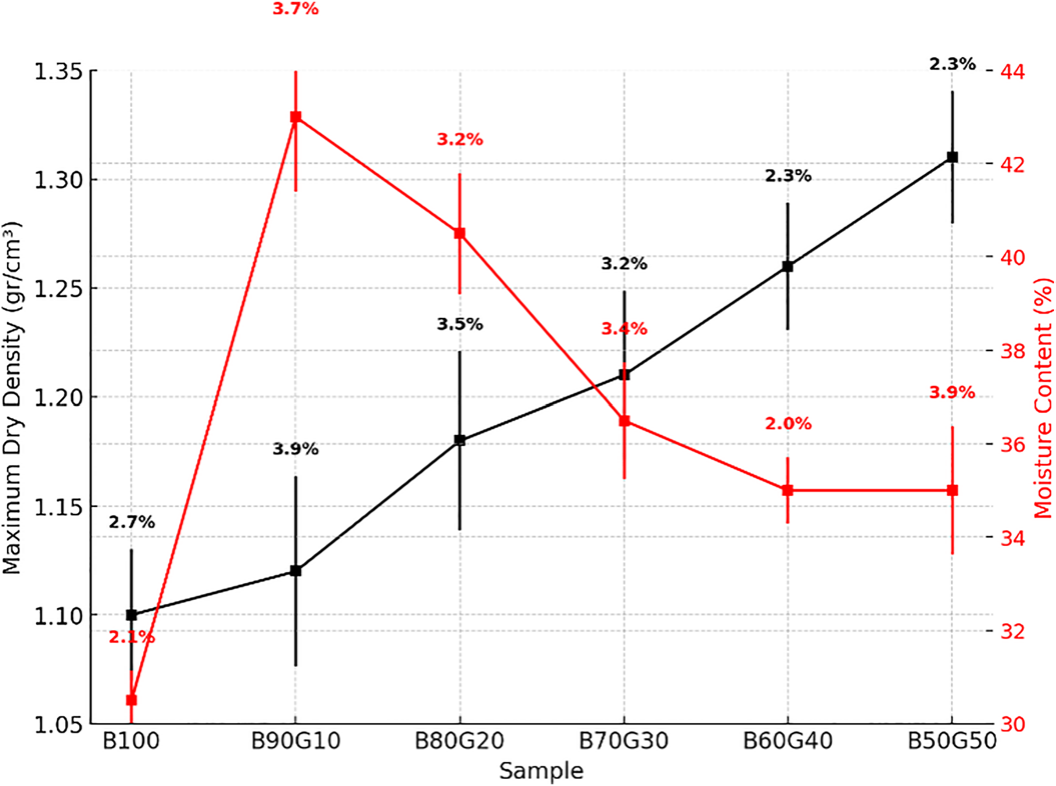
Optimum moisture content and maximum dry density from the standard compaction of the six mixtures.

To evaluate the compressive strength of the mixtures, cohesion and friction angle are critical factors, as shown in Fig. 5. Cohesion decreases as the bentonite proportion reduces, while the friction angle significantly increases with higher goethite content due to its surface roughness. SEM images comparing B100 and B50G50 reveal the cohesive properties of bentonite and frictional properties of goethite particles. In the B70G30 mixture, the strong cohesion and connectivity of bentonite, combined with goethite’s frictional effects, enhance shear strength, resulting in the highest uniaxial compressive strength. Additionally, Fig. 5 illustrates that maximum dry density increases with goethite content due to its denser nature replacing bentonite, while optimum moisture content generally decreases because of goethite’s lower water absorption compared to bentonite.

**Fig. 6:**
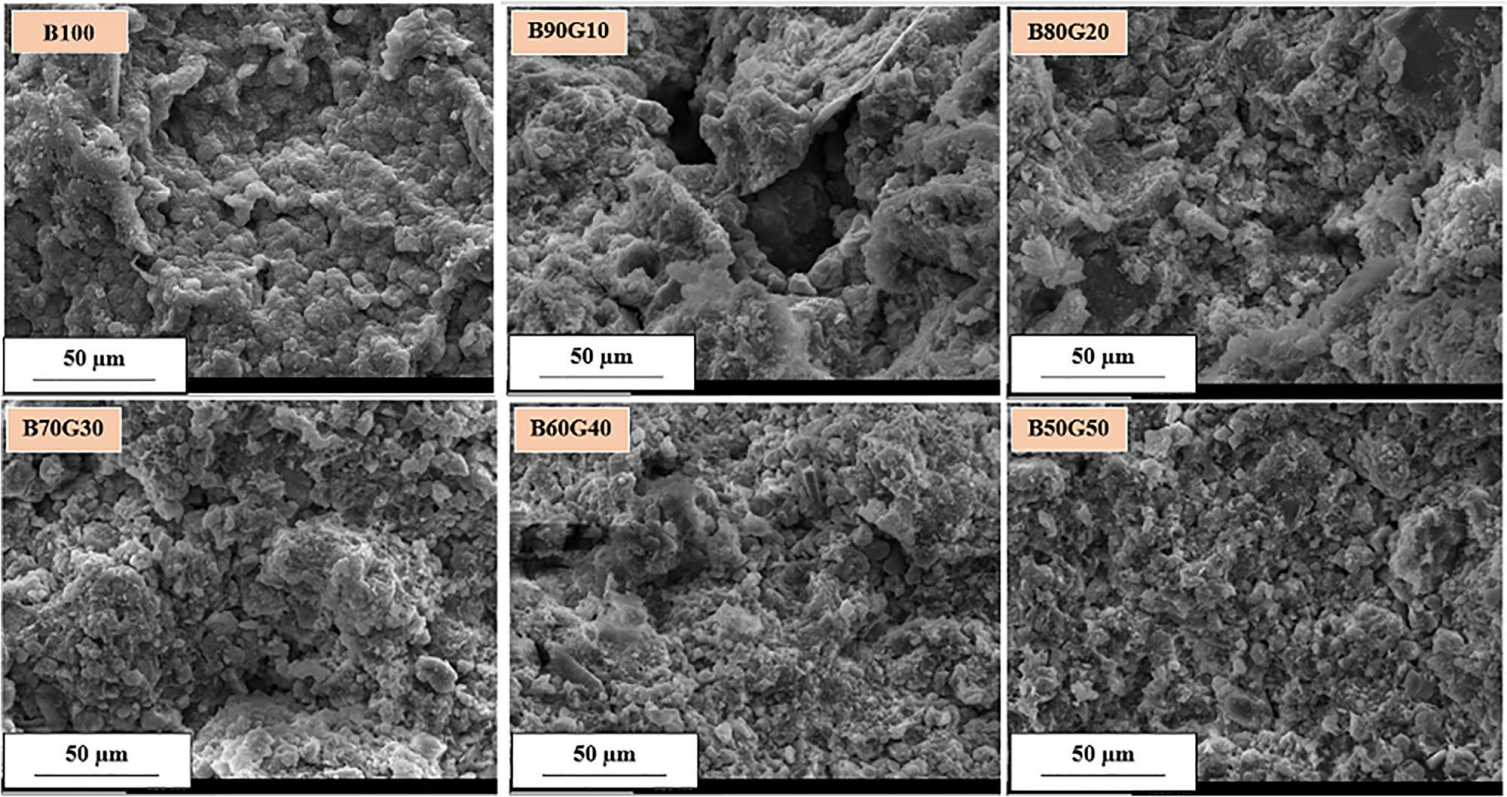
The SEM images of the studied mixtures.

Figure 6 illustrates the SEM images of the mixtures, which present that in the mixture with 10 percent goethite, the heterogeneity and void spaces are increased, requiring excess water content to reach the maximum dry density compared to pure bentonite. As the proportion of goethite increases, the homogeneity gradually increases, resulting in a reduction in the optimum moisture content. Table 4 presents the elemental analyses of bentonite and studied mixtures, which would be implemented for further radiation shielding investigations.

**Table 4:** Elemental analysis results for bentonite–goethite mixtures by SEM (in percentage).

Element	Bentonite	B90G10	B80G20	B70G30	B60G40	B50G50
O	45.72	45.988	46.256	46.524	46.792	47.06
Fe	11.92	14.054	16.188	18.322	20.456	22.59
Ca	7.41	8.06	8.71	9.36	10.01	10.66
Si	14.79	13.902	13.014	12.126	11.238	10.35
Na	10.375	9.408	8.441	7.474	6.507	5.54
Al	3.315	3.204	3.093	2.982	2.871	2.76
Mg	0.9	0.918	0.936	0.954	0.972	0.99
Cl	5.57	4.464	3.358	2.252	1.146	0.04

### Theory and methods of radiation shielding

Gamma-ray is an electromagnetic radiation that is emitted from the transitions and interactions that occur within an excited atom. Gamma rays have no electric charge or mass and therefore have the ability to penetrate more materials than alpha and beta particles. The energy range of gamma rays is from 124 keV to 24.1 MeV. Despite its various applications in different industries such as medicine, agriculture, technology, etc., gamma rays can cause irreparable damage when exposed to living organisms. The amount of gamma-ray absorption, which is determined by the linear attenuation coefficient, is estimated using laboratory, simulation, and theoretical approaches. Based on radiation shielding theory, the Beer-Lambert law explains the reduction in radiation intensity as it passes through a shielding material. The attenuation coefficient ($\mu$) is calculated according to this law for a shielding material with a constant thickness that is homogeneous and isotropic using Eq. (1).

1 $$\begin{aligned} I = I_{0}e^{- \mu t} \end{aligned}$$

Where gamma-ray intensity with and without a barrier is represented by *I* and $I_0$, and t is the thickness of the barrier. Other important factors to consider when assessing radiation shielding materials are the half-value layer (HVL) and tenth-value layer (TVL). These values represent the required thicknesses of the shielding material to reduce gamma-ray intensity to 50% and 10%, respectively. According to Edmund (2021), HVL and TVL can be calculated using Eqs. 2 and 3^35^.

2 $$\begin{aligned} & HVL = \frac{Ln(2)}{\mu }(m) \end{aligned}$$

3 $$\begin{aligned} & TVL = \frac{Ln(10)}{\mu }(m) \end{aligned}$$

**Fig. 7:**
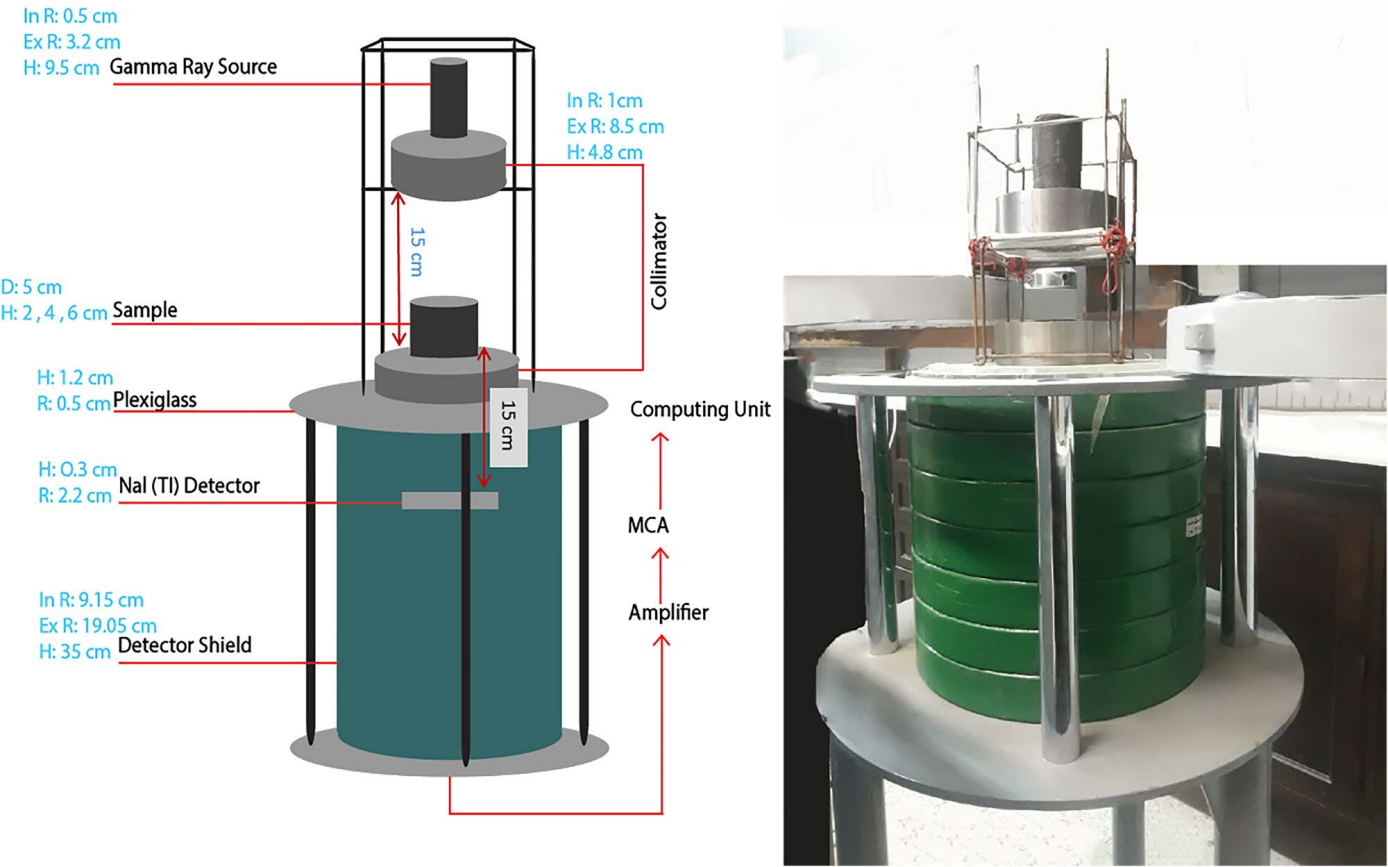
The linear attenuation measurement experimental setup using Na(Tl) detector.

The experimental setup is depicted in Fig. 7, which consists of a radiation source, collimator, amplifier, multi-channel analyzer (MCA), and a computing unit. To attain the appropriate narrowness of the irradiation, two steel collimators are employed at the top and bottom of the specimen. The radiation source of Co$^{60}$ is employed to emit gamma rays with energies of 661.6, 1173.2, and 1332.5 keV as the three commonly used energy levels^36–38^. The source–sample and sample–detector distances were both 15 ± 0.1 cm. Steel collimators, 4.8 ± 0.1 cm thick with a 2 ± 0.1 cm diameter central aperture, were used; the lower one sat on a plexiglass sheet above the detector shield, and the upper one was aligned with a steel frame. The radiation source was placed above the upper collimator’s aperture. Samples, with thicknesses of 2, 4, or 6 ± 0.1 cm and a 5 ± 0.1 cm diameter, were placed flush on the lower collimator’s aperture. The sample diameter did not affect the linear attenuation coefficient calculation. The MCNP model matched the experimental setup, with a detector count rate of $\approx$ 25,000 counts/s and regular constancy checks for stability.

Gamma rays are radiated to the sample, and the intensity of the rays before ($I_0$) and after (*I*) the sample is detected using the Na(Tl) detector. To examine the experimental measurement error, the error propagation is derived based on Eq. (4):

4 $$\begin{aligned} \Delta (\mu )= \frac{1}{t}\sqrt{\left( \ln \left( \frac{I_{0}}{I} \right) \right) ^{2}\left( \frac{\mathrm {\Delta }t}{t} \right) ^{2} + {(\frac{\mathrm {\Delta }I_{0}}{I_{0}})}^{2} + {(\frac{\mathrm {\Delta }I}{I})}^{2}} \end{aligned}$$

where, $\Delta t$, $\Delta I_0$, and $\Delta I$ are the measurement accuracy of time (*t*), $I_0$, and, *I* respectively. Moreover, the linear attenuation coefficient for the desired materials was also simulated employing Monte Carlo N-Particle Transport Code (MCNP) and then assessed with the use of the XCOM and PHY-X databases.

Methodology-wise, MCNP uses Monte Carlo simulations for detailed radiation transport modeling, whereas Photon Cross Sections Database (XCOM) and Physics of X-rays (Phy X) databases rely on pre-calculated linear attenuation coefficients based on empirical data. Moreover, in terms of flexibility, MCNP can manage complex geometries and material variations, while the databases are quicker but less adaptable. Regarding detail, MCNP provides in-depth interaction insights, whereas databases offer generalized average values without considering complex geometries.

**Fig. 8:**
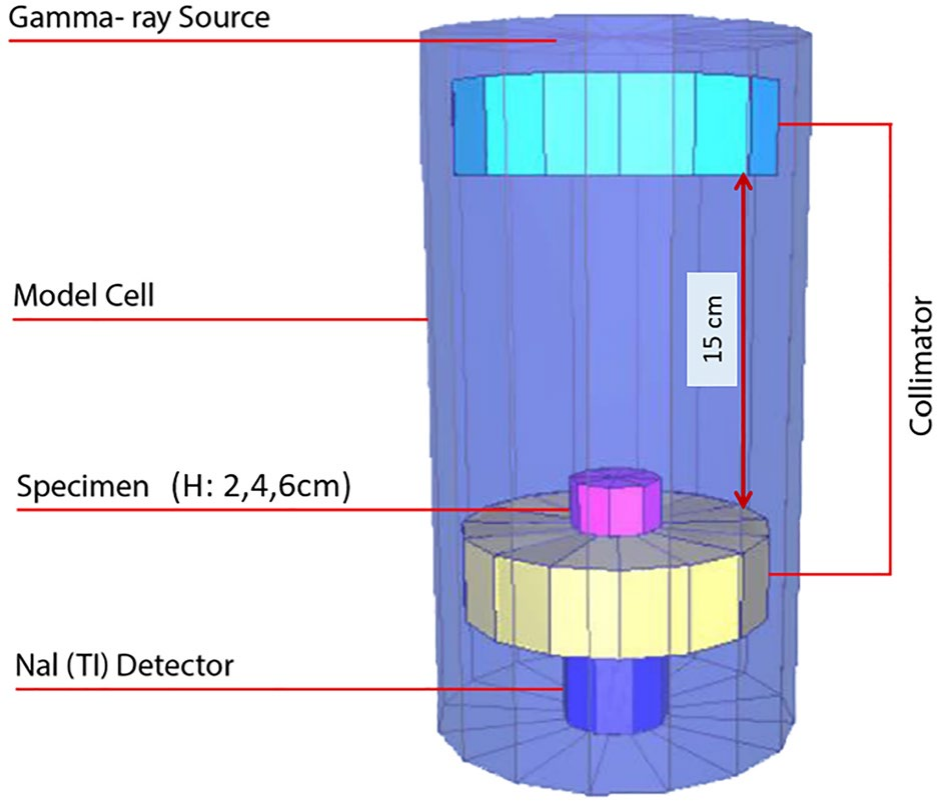
The three-dimensional view of the MCNP models.

To simulate the radiation shielding in the MCNP code, individual cells were established for each material, and their properties were allocated by considering their specific weight, surface area and height of the sample, radiation energy levels, percentages of chemical elements, and their atomic and atomic mass number. Figure 8 illustrates the three-dimensional view of the MCNP models.

After assigning the component properties to each cell, the energy levels of the radiation source are assigned similarly to the laboratory setup. Then, a group of rays is directed randomly toward the sample, and scattering, attenuation, and absorption states are detected. This process is carried out both with and without samples to obtain the gamma-ray intensities, represented by *I* and $I_0$, respectively. The linear attenuation coefficient ($\mu$) is then computed utilizing the Lambert-Beer law (Eq. 1). The advantage of this method compared to laboratory tests is the possibility of obtaining results at any desired energy level in safer, less time-consuming, and cost-effective conditions.

The XCOM and PHY-X databases are utilized to estimate photon cross-sections for a variety of scattering, photoelectric absorption, and total attenuation coefficients for elements, compounds, and mixtures across a range of energies from 1 keV to 100 GeV. The total attenuation coefficients for mixtures and compounds are determined by the summation of the corresponding atomic constituent quantities. To perform calculations in the XCOM database, the percentage of elements in the compound and their atomic number are required. However, in the PHY-X database, in addition to the percentage of elements in the compound and their atomic number, the density of the material must be determined.

### Hydro-mechanical properties experiments

Unconfined compressive strength, shear strength, and hydraulic permeability are the three geomechanical parameters that are assessed to ensure the stability and conductivity of landfill liners^39–45^. The unconfined compressive strength (UCS) tests were performed three times for each mixture in accordance with ASTM D2166/D2166M-16 on specimens compacted at their optimum moisture content and maximum dry density determined from the standard Proctor test. Cylindrical samples with a height-to-diameter ratio of approximately 2:1 (76 mm $\times$ 38 mm) were axially loaded at a constant strain rate of 1% per minute until failure. The direct shear tests were conducted following ASTM D3080/D3080M-11 using samples compacted to the same maximum dry density and optimum moisture content as the UCS specimens. Normal stresses of 40, 70, 100 and 130 kPa were applied, and shearing was carried out at a constant displacement rate of 0.5 mm/min under drained conditions.

**Fig. 9:**
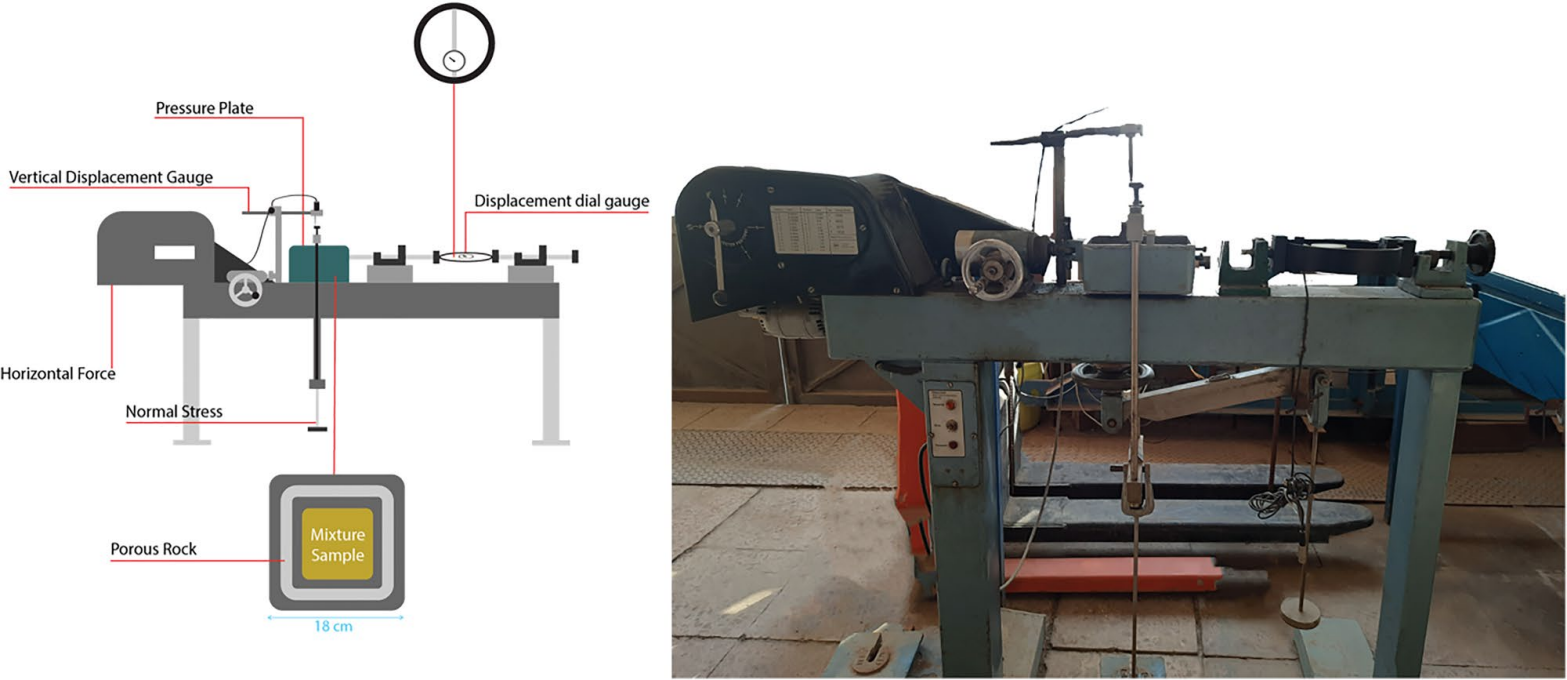
Actual and schematic setup of direct shear test.

**Fig. 10:**
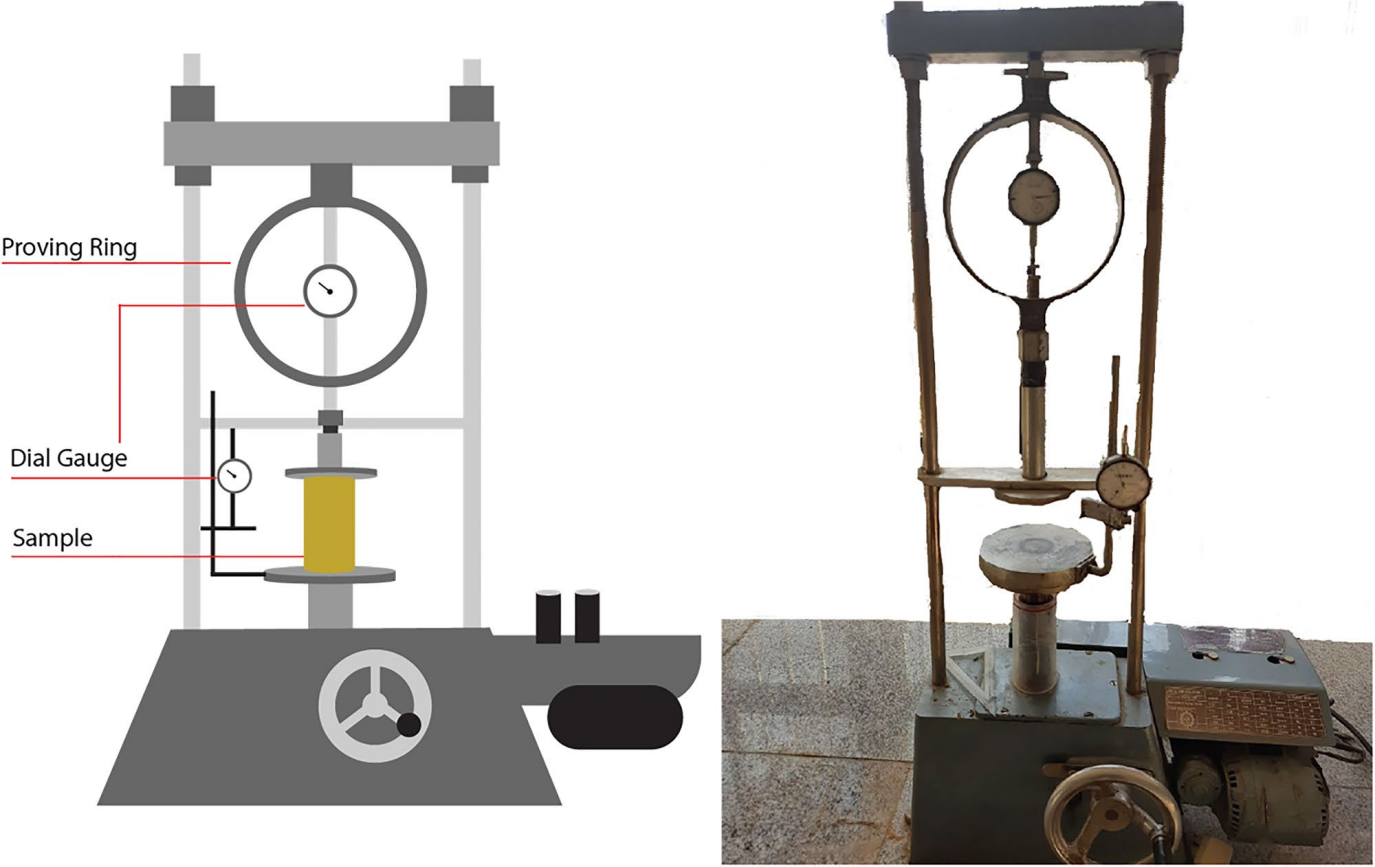
Actual and schematic setup of unconfined compressive strength test.

Moreover, for each test, three samples are prepared and examined for an individual mixture, and the results are presented as the mean values. Figures 9 and 10 illustrate the schematic and actual test setup for the unconfined compressive strength test and direct shear test, respectively.

Based on EPA regulations, the hydraulic permeability of low-level radioactive waste (LLRW) landfill liners should be less than 1$\times$10$^{-9}$ m/s to protect the surrounding soil and water contamination^2,46^. The implemented ASTM D5856-95 “Standard Test Method for Measurement of Hydraulic Conductivity of Porous Material Using a Rigid-Wall, Compaction-Mold Permeameter” is normally used for compacted mixtures with hydraulic conductivity less than 1$\times$10$^{-5}$ m/s. The prepared mixtures are first compacted under maximum dry density and optimal moisture content, with void ratio of 1.45, 1.52, 1.53, 1.57, 1.61, 1.66 for B100, B90G10, B80G20, B70G30, B60G40, and B50G50, respectively. Where the temperature is set to be in the range of 20 ± 2$^{\circ }$C and the tests are performed three times for each sample mixture.

**Fig. 11:**
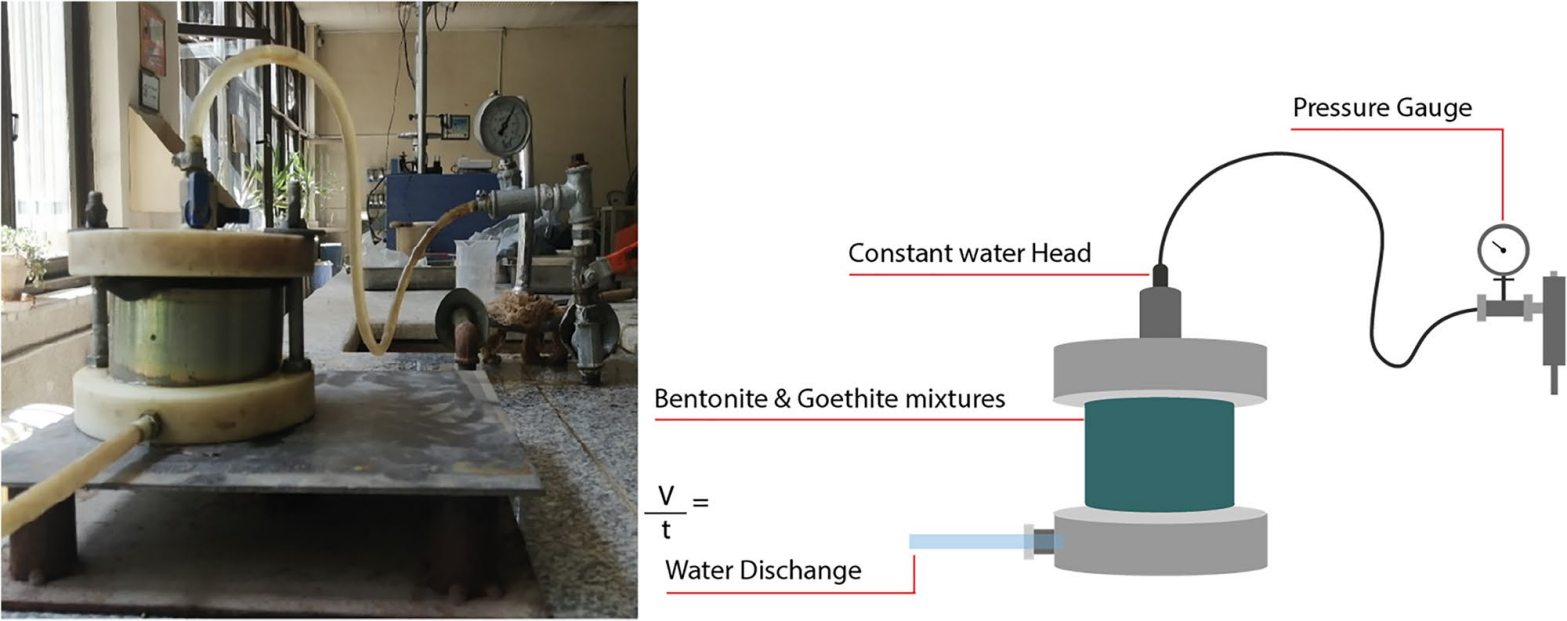
Actual and schematic setup of the hydraulic conductivity test.

Due to the very low permeability of sodium bentonite, a water pressure head of 300 kPa was implemented over the samples. Figure 11 presents the hydraulic conductivity test setup. Eq. 5 is employed to calculate the hydraulic conductivity of the sample mixtures ($\kappa$):

5 $$\begin{aligned} \kappa = \frac{Q \times L}{A \times h \times t} \end{aligned}$$

where *Q* is the discharge volume (m$^3$), *L* is the specimen length (m), *A* is the specimen area (m$^2$), *h* is the water head (m), and *t* stands for time (s).

## Results

This section presents a comprehensive analysis of the experimental and simulation results concerning the radiation shielding capacity and hydro-mechanical properties of bentonite-goethite mixtures. The objective is to identify an optimal composition that provides effective radiation attenuation while maintaining acceptable strength and permeability standards for landfill liner applications.

### Radiation shielding performance

To evaluate the radiation shielding performance of the bentonite–goethite mixtures, laboratory experiments, MCNP simulations, and reference databases (XCOM and PHY-X) were employed. The linear attenuation coefficient ($\mu$) was determined at three gamma-ray energies: 661.6, 1173.2, and 1332.5 keV. Table 5 presents the $\mu$ values from experimental measurements using an NaI(Tl) detector, along with calculated errors, simulation outputs, and database values. The required liner thicknesses for reducing gamma-ray intensity to 50% (HVL) and 10% (TVL) are shown in Table 6. The results from all three approaches exhibit strong agreement, confirming the reliability of both the experimental setup and simulation methodology.

**Fig. 12:**
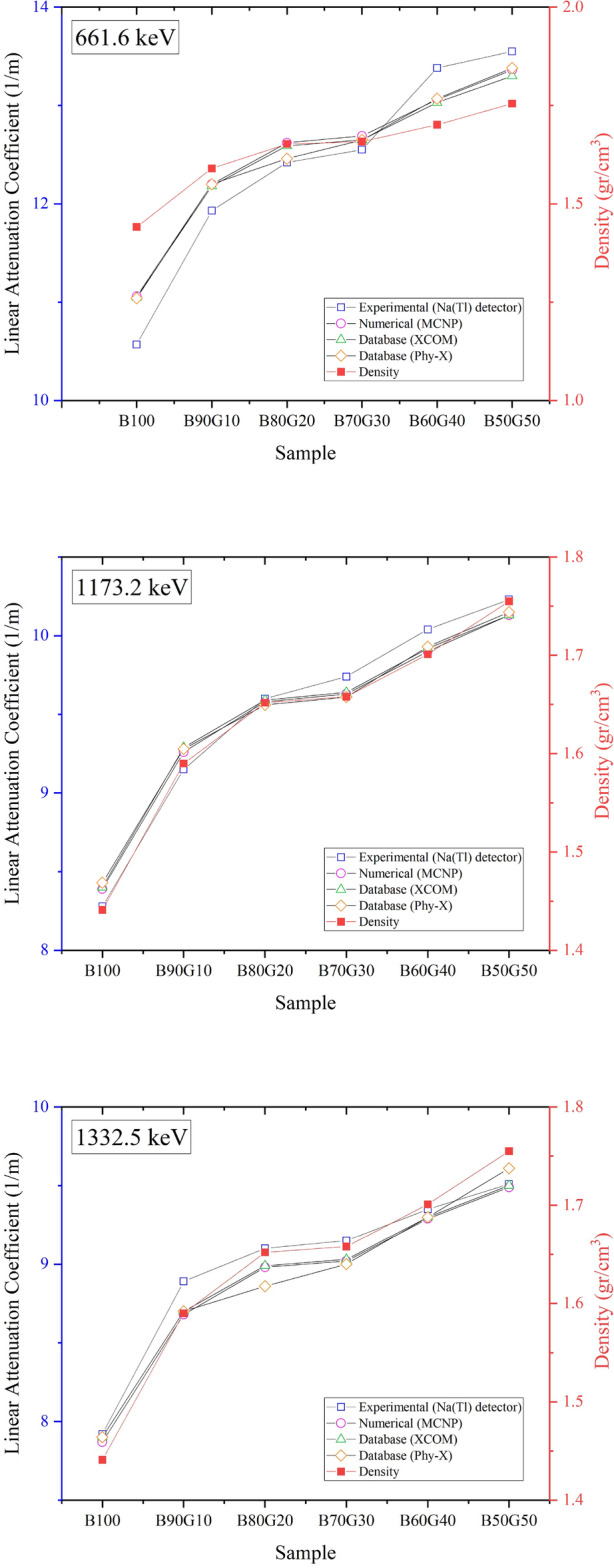
Densities and linear attenuation coefficients were obtained at energy levels of 661.6 keV, 1173.2 keV, and 1332.5 keV.

Figure 12 compares $\mu$ as a function of goethite content across all energy levels, alongside the density variation of the mixtures. The trends confirm the consistency between experimental, simulation, and database results (with MCNP represented by circles, and XCOM and PHY-X by triangles and diamonds, respectively). Minor deviations, such as in B100 at 1332.5 keV, likely arise from sample heterogeneity or detector calibration limits but remain within the acceptable error margins ($\Delta \mu$ in Table 5).

**Table 5:** The linear attenuation coefficient values measured and calculated from experimental, simulation, and standard reference database.

Factor	$\mu (1/m)$														
	661.6 (keV)					1173.2 (keV)					1332.5 (keV)				
Mixture	Experimental		MCNP	XCOM	PHY-X	Experimental		MCNP	XCOM	PHY-X	Experimental		MCNP	XCOM	PHY-X
	$\mu$	$\Delta (\mu )$				$\mu$	$\Delta (\mu )$				$\mu$	$\Delta (\mu )$			
B100	10.57	0.2	11.06	11.05	11.04	8.28	0.24	8.39	8.4	8.43	7.92	0.55	7.87	7.9	7.9
B90G10	11.93	0.35	12.2	12.18	12.2	9.15	0.25	9.26	9.29	9.28	8.89	0.31	8.68	8.7	8.7
B80G20	12.42	0.25	12.62	12.59	12.46	9.6	0.21	9.58	9.59	9.56	9.1	0.22	8.98	8.99	8.86
B70G30	12.55	0.3	12.69	12.65	12.65	9.74	0.65	9.63	9.64	9.61	9.15	0.54	9.02	9.03	9
B60G40	13.38	0.32	13.06	13.03	13.07	10.04	0.39	9.9	9.92	9.93	9.35	0.24	9.29	9.3	9.3
B50G50	13.55	0.36	13.36	13.3	13.38	10.23	0.46	10.13	10.13	10.15	9.51	0.53	9.49	9.5	9.61

**Table 6:** The required liner thicknesses of the mixtures to reduce gamma-ray intensity to 50% (HVL) and 10% (TVL).

Sample	Energy level					
	661.6 (keV)		1173.2 (keV)		1332.5 (keV)	
	HVL (m)	TVL (m)	HVL (m)	TVL (m)	HVL (m)	TVL (m)
B100	0.07	0.22	0.08	0.28	0.09	0.29
B90G10	0.06	0.19	0.08	0.25	0.08	0.26
B80G20	0.06	0.19	0.07	0.24	0.08	0.25
B70G30	0.06	0.18	0.07	0.24	0.08	0.25
B60G40	0.05	0.17	0.07	0.23	0.07	0.25
B50G50	0.05	0.17	0.07	0.23	0.07	0.24

The results show a clear positive correlation between $\mu$ and goethite content, in line with the theoretical relationship $\mu \propto \rho$. For instance, at 661.6 keV, $\mu$ increased from 10.57 m$^{-1}$ for pure bentonite (B100) to 13.55 m$^{-1}$ for B50G50—a 28.2% improvement. At 1173.2 keV and 1332.5 keV, $\mu$ rose from 8.28 to 10.23 m$^{-1}$ (23.6% increase) and from 7.92 to 9.51 m$^{-1}$ (20.1% increase), respectively. These gains closely parallel the increase in dry density from 1.38 g/cm$^{3}$ (B100) to ~1.62 g/cm$^{3}$ (B50G50), driven by goethite’s high density (4.3 g/cm$^{3}$). This parallelism is evident in Figure 12, where density (black squares) and $\mu$ progress are almost identical across all energy levels.

Interestingly, the $\mu$–goethite relationship is nonlinear. The steepest increase occurs at low goethite additions (0–10%), suggesting that small amounts significantly enhance attenuation due to the dominance of high atomic number elements (e.g., Fe) in photon interactions. Between 10–30%, the rate of increase slows, indicating a saturation effect. A secondary rise is observed from 30–50%, possibly due to denser packing or synergistic effects between bentonite and goethite microstructures. Averaged over these ranges, $\mu$ increases by $\sim$12%, $\sim$4%, and $\sim$6%, respectively.

In practical terms, higher goethite content improves shielding efficiency by reducing HVL and TVL, enabling thinner liners without compromising protection. For example, at 661.6 keV, HVL decreased from 0.07 m (B100) to 0.05 m (B50G50)—a 28.5% reduction—suggesting potential cost and material savings in large-scale applications. However, diminishing returns beyond 30% replacement highlight the need to optimize mixtures based on specific energy-level requirements.

Overall, the integration of experimental, computational, and theoretical analyses provides robust validation of goethite-enhanced bentonite as a high-performance, space-efficient radiation shielding material. Figure 12 serves as a concise visual synthesis of these findings, capturing the interplay between composition, density, and attenuation performance.

### Shear strength behavior

**Fig. 13:**
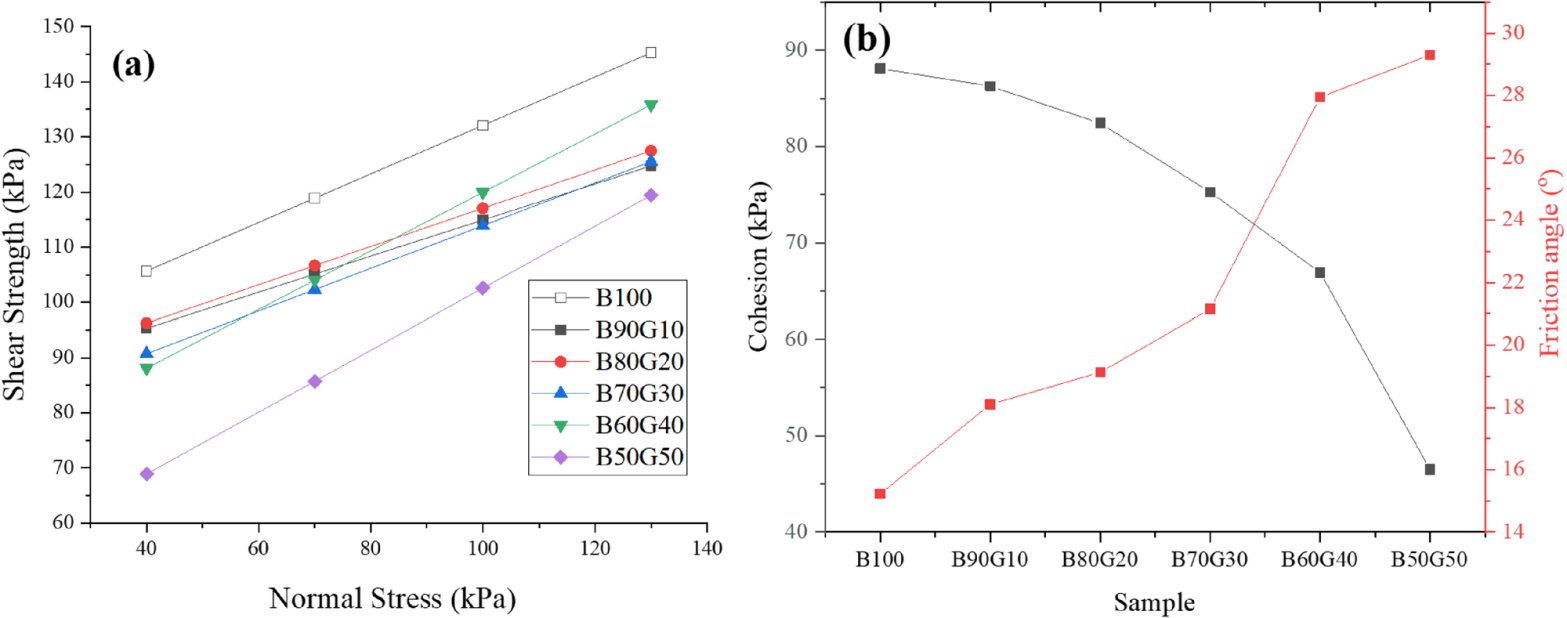
(**a**) The shear strength of the mixtures against normal stress based on the Mohr–Coulomb failure criterion (**b**) and obtained cohesion––black line - and friction angle—red line.

The shear strength of the mixtures was assessed to assess their stability in landfill applications and to mitigate the risk of potential slope failures in these soil-based structures. To achieve this, direct shear tests were conducted at four normal stress levels: 40, 70, 100, and 130 kPa, as illustrated in Fig. 13a. Based on the Mohr–Coulomb failure criterion, the slope of the resulting failure envelopes indicates the friction angle, while the intercept at the origin represents the cohesion of the soil mixtures, both of which are detailed in Fig. 13b.

The results demonstrate a strong reduction in cohesion, from 28 kPa for pure Bentonite (B100) to approximately 14 kPa for B50G50 mixture —representing a 50% decrease (Fig. 13b). Conversely, the friction angle increased markedly from 20.4$^{\circ }$ to 43.2$^{\circ }$, an increase of more than 110%. This trend reflects a clear transition from a cohesion-controlled to a friction-controlled behavior as goethite content increases, likely due to the rougher, angular surface morphology of goethite particles enhancing interparticle friction.

From a design perspective, these findings have two key implications. Reduced cohesion may compromise slope stability under low confining pressures, making the liner more susceptible to shear failure in certain scenarios. However, the substantial increase in friction angle can enhance resistance to lateral movement, especially in composite or layered landfill liner systems. These opposing effects underscore the need for site-specific design adjustments based on expected loading conditions, slope geometry, and the intended performance criteria of the waste containment barrier.

### Uniaxial compressive strength (UCS)

**Fig. 14:**
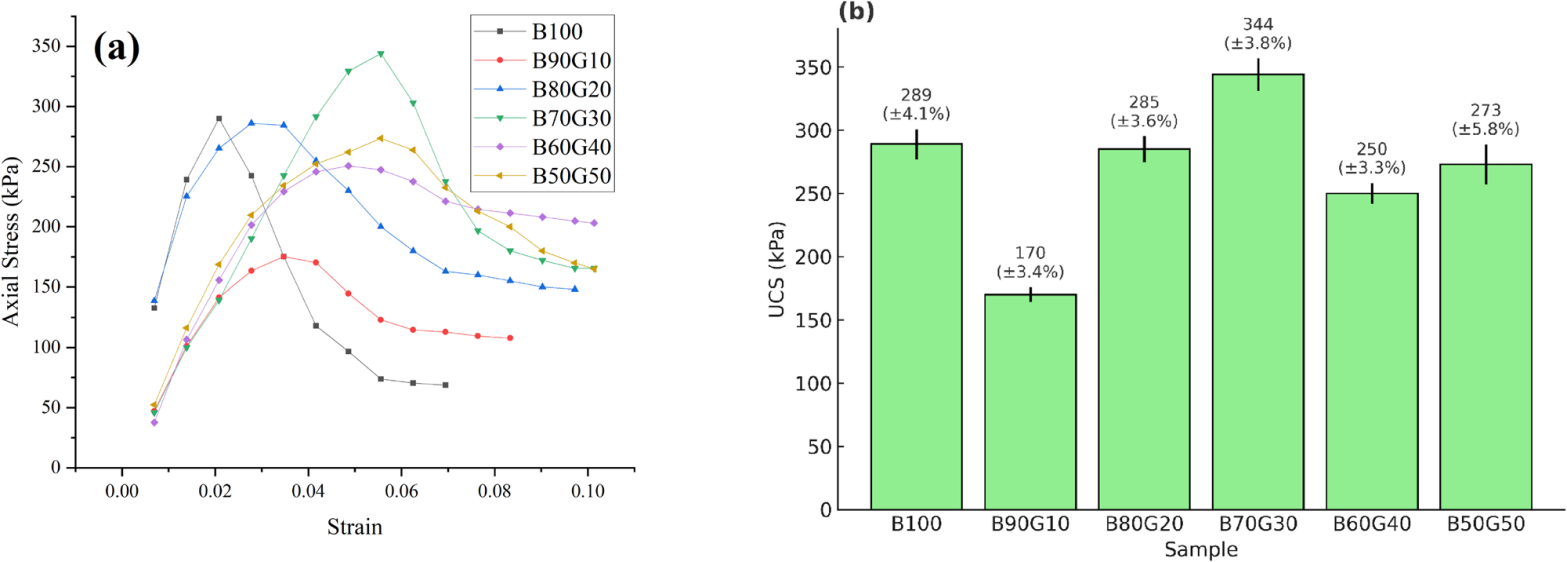
Stress-strain curve obtained from (**a**) axial stress against strain and (**b**) the uniaxial compressive test for the six specimens.

To evaluate the uniaxial compressive strength of the mixtures, UCS tests were conducted in triplicate for each composition, and the average results were used for analysis. The corresponding stress-strain curves and peak UCS values are illustrated in Fig. 14.

The results reveal a non-monotonic relationship between the goethite content and compressive strength. For pure bentonite (B100), UCS was measured at 289 kPa, which is in a good agreement with the previous studies^47,48^. The addition of 10% goethite (B90G10) introduced microstructural heterogeneity and larger void spaces—as observed in SEM images (Fig. 6)—resulting in a significant 40% decrease in UCS to approximately 170 kPa. Adding 10% goethite makes the mixture less cohesive, and the extra friction is not sufficient to overcome it. As a result, the UCS—which primarily relies on internal friction in the absence of confining stress—experiences a significant reduction.

As the goethite content increased to 30% (B70G30), the microstructure became more uniform and densely packed, reducing voids and enhancing homogeneity. This resulted in the highest UCS value of 344 kPa—about 20% higher than that of pure bentonite—indicating an optimal balance between cohesion strength and internal frictional resistance. This peak coincides with the intersection point of cohesion and friction angle trends shown in Fig. 14b, suggesting maximum internal bonding and shear resistance at this composition. However, further increasing goethite content beyond 30% results in a decline in UCS, with B50G50 dropping to approximately 273 kPa. This reduction is likely due to continued cohesion loss outweighing the benefits of increased internal friction. Despite the decrease in peak strength, the residual strength after failure generally increased with higher goethite content—from approximately 75 kPa (B100) to 175 kPa (B50G50)—as increased friction angles played a more dominant role in the post-failure behavior (Fig. 14a).

### Hydraulic conductivity

**Fig. 15:**
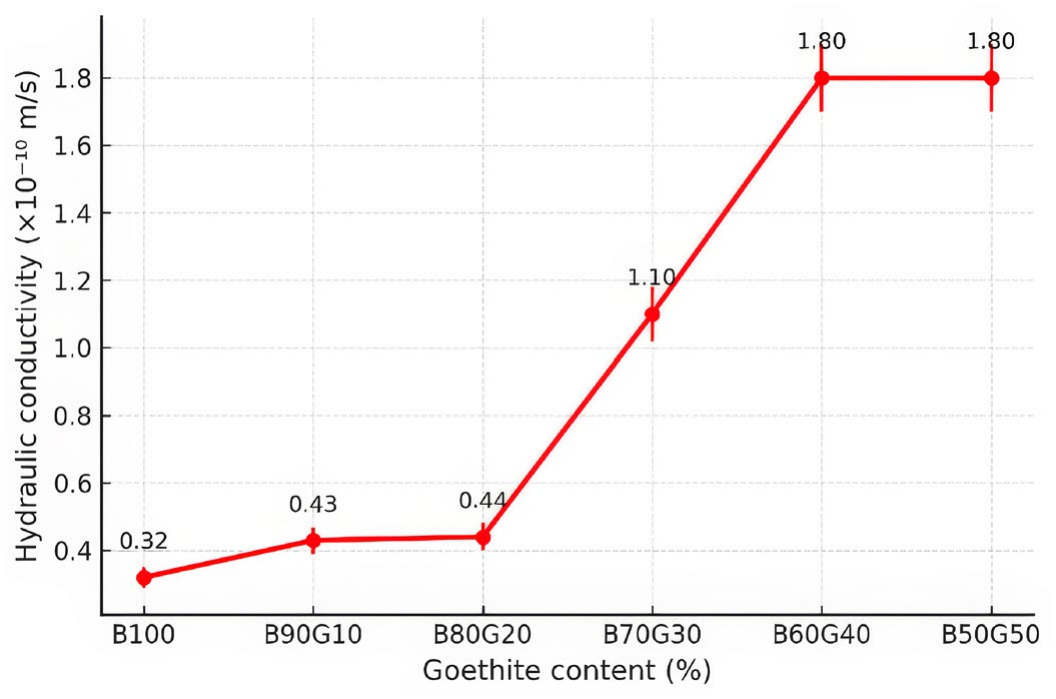
Hydraulic conductivity of the mixtures and the permissible value based on EPA standard.

In this research, hydraulic conductivity tests were performed based on the mentioned procedure. Figure 15 shows the hydraulic permeability of the bentonite-goethite mixtures. Generally, hydraulic conductivity increased with higher goethite content, which can be explained by the lower proportion of bentonite, a relatively impermeable and swelling material.

The measured hydraulic conductivity increased from 6.7 $\times 10^{-11}$ m/s for pure bentonite (B100) to 1.8 $\times 10^{-10}$ m/s for B50G50, where the B70G30 is comparable with the geosynthetic clay liner (GCL) representing 1.2$\times 10^{-10}$ m/s^49^. This threefold increase reflects the progressive reduction of bentonite content, which is responsible for fine particle sealing and swelling behavior. Despite this increase, permeability remained below the EPA threshold of 1.0 $\times 10^{-9}$ m/s for all studied mixtures.

A sharp increase in the hydraulic conductivity was observed between B30G70 and B40G60 mixtures, suggesting a critical reduction in sealing effectiveness. Consequently, designers should note that while B70G30 mixture offers superior mechanical and radiation shielding performance, mixtures exceeding 40% goethite content should be used with caution due to potential permeability concerns. To summarize the obtained results for all studied mixtures, the hydro-mechanical properties are presented in Table 7.

**Table 7:** The obtained results for hydro-mechanical tests.

Sample	$\kappa$ ($\times 10^{-10}$ m/s)	C (kPa)	$\phi$	UCS (kPa)
B100	0.32 ±3.43	88.1	15.2	289
B90G10	0.43 ±4.23	85.3	18.1	170
B80G20	0.44 ±5.93	82.4	19.1	285
B70G30	1.10 ±4.41	75.3	21.2	344
B60G40	1.80 ±4.64	66.9	28.0	250
B50G50	1.80 ±3.66	46.5	29.3	273

## Discussion

In this study, experimental measurements, simulations, and databases are employed to investigate the radiation shielding performance of bentonite–goethite mixtures. The findings highlight the potential of these mixtures in improving radiation attenuation while maintaining the essential hydro-mechanical properties required for hazardous waste landfill liners. The approach offers a sustainable solution by utilizing waste materials from mineral processing, contributing to environmental sustainability and waste management practices.

The bentonite-goethite mixtures demonstrate significant potential as landfill liners, balancing enhanced radiation shielding with hydro-mechanical performance. With increasing goethite content, the SEM images reveal improved particle packing and reduced pore spaces, indicating better compaction and higher density. This enhancement in microstructural uniformity not only lowers the optimum moisture content but also contributes to an increased linear attenuation coefficient, as higher density materials provide more effective radiation attenuation.

The linear attenuation coefficient ($\mu$) increased by 20.1–28.2% with goethite content up to 50% (B50G50), driven by goethite’s high density (4.3 g/cm$^3$) and iron content, reducing HVL and TVL by up to 28.5% for thinner, cost-effective liners. However, the nonlinear $\mu$–goethite relationship, with diminishing returns beyond 30%, suggests an optimal range (e.g., B70G30) for shielding efficiency. Shear strength tests revealed a trade-off: cohesion decreased by 50% (28 to 14 kPa), while friction angle increased 110% (20.4$^{\circ }$ to 43.2$^{\circ }$), enhancing resistance to lateral movement but risking slope stability under low confining pressures. The B70G30 mixture achieved the highest UCS (870 kPa, 20.8% above pure bentonite) due to optimal cohesion-friction balance, though hydraulic conductivity increased threefold ($6.7\times 10^{-10}$ to $1.8\times 10^{-10}$ m/s), remaining within EPA limits ($1.0\times 10^{-9}$ m/s).

The B70G30 composition emerges as a sustainable, high-performance option, leveraging waste-derived goethite to enhance shielding and strength while meeting permeability standards. However, limitations such as sample heterogeneity, static testing conditions, and a narrow gamma-ray energy range (661.6–1332.5 keV) suggest the need for refined mixing techniques, dynamic testing, and broader energy assessments. Future research should also evaluate long-term durability under environmental stresses and the economic feasibility of large-scale goethite use. These findings align with studies on clay-additive liners^50,51^, supporting the adoption of goethite-enhanced bentonite for hazardous waste containment with tailored design to address cohesion and permeability trade-offs.

In addition to the technical results, from a sustainability perspective, reusing waste-derived goethite significantly reduces both material cost and embodied energy compared with sourcing and processing virgin mineral additives. This reuse supports the circular economy by converting mining waste into a functional and low-impact engineering material.

## Conclusion

This study examines the use of goethite, a mining waste by-product, as an additive to bentonite for developing new engineered barrier materials in hazardous waste landfill liners, with emphasis on their radiation shielding and hydro-mechanical behavior.

A comprehensive evaluation was conducted through experimental testing, validated numerical simulation (MCNP), and standard reference database (XCOM and PHY-X), considering various goethite addition levels of 10, 20, 30, 40, and 50.

The following are the most important conclusions achieved:

- Radiation shielding capacity increases markedly with higher goethite content, reflected by consistent improvements in the linear attenuation coefficient at all tested energies (661.6, 1173.2, and 1332.5 keV), closely tied to increases in dry density. Experimental results showed strong agreement with numerical (MCNP) and database predictions (XCOM, PHY-X), confirming the reliability of these tools for evaluating and predicting shielding performance in similar material systems.
- Shear strength behavior shows a clear trade-off with increasing goethite: cohesion decreases by up to 50%, while the friction angle more than doubles due to the shift from a cohesion-dominated to a friction-dominated microstructure driven by the angularity and roughness of goethite particles. Correspondingly, UCS exhibits a non-monotonic trend with a peak at B70G30, where the reduced bentonite content lowers cohesion but the increased goethite enhances frictional resistance, indicating that mechanical strength can be optimized by balancing cohesion from bentonite and frictional resistance from goethite.
- Although all mixtures are in the allowable range of hydraulic conductivity according to EPA regulation, different trends are observed increasing the goethite content where the obtained values are comparable with those from previous studies implementing GCL or various additives. A 28% increment is observed from B100 to 20% goethite in the mixtures, whereas for 40% of the goethite proportion, it upsurges three times and stays constant in higher goethite percentages.

Three objectives, including linear attenuation coefficient, mechanical strength, and hydraulic permeability, are considered to evaluate the performance of the studied mixtures. The results show that growing the goethite proportion increases the linear attenuation coefficient. Among the tested mixtures, B70G30 demonstrates the best overall performance, achieving the highest uniaxial compressive strength due to an ideal balance of decreasing cohesion and increasing friction angle. Although hydraulic permeability rises with higher goethite content, all samples, including B70G30, meet EPA standards, confirming it as the optimal composition for structural integrity and shielding efficiency. Therefore, the B70G30 would be the best candidate as a cover layer for low level radioactive wastes disposals.

## Data Availability

The original contributions presented in the study are included in the article; further inquiries can be directed to the corresponding author
